# Thermal activation of ‘allosteric-like’ large-scale motions in a eukaryotic Lactate Dehydrogenase

**DOI:** 10.1038/srep41092

**Published:** 2017-01-23

**Authors:** Marina Katava, Marco Maccarini, Guillaume Villain, Alessandro Paciaroni, Michael Sztucki, Oxana Ivanova, Dominique Madern, Fabio Sterpone

**Affiliations:** 1 Laboratoire de Biochimie Théorique, IBPC, CNRS UPR9080, Univ. Paris Diderot, Sorbonne Paris Cité, 13 rue Pierre et Marie Curie, 75005, Paris, France; 2 Univ. Grenoble Alpes - Laboratoire TIMC/IMAG UMR CNRS 5525, Grenoble Pavillon Taillefer Domaine de la merci, 38700 La Tronche, France; 3 Dipartimento di Fisica e Geologia, Universitá di Perugia, via A. Pascoli, 06123 Perugia, Italy; 4 European Syncrotron Radiation Facility, 6, rue Jules Horowitz, 38042, Grenoble, France; 5 Jülich Centre for Neutron Science (JCNS) at Heinz Maier-Leibnitz Zentrum (MLZ), Forschungszentrum Jülich GmbH, Garching, Germany; 6 Institut de Biologie Structurale (IBS), Univ. Grenoble Alpes, CEA, CNRS, 38044 Grenoble, France

## Abstract

Conformational changes occurring during the enzymatic turnover are essential for the regulation of protein functionality. Individuating the protein regions involved in these changes and the associated mechanical modes is still a challenge at both experimental and theoretical levels. We present here a detailed investigation of the thermal activation of the functional modes and conformational changes in a eukaryotic Lactate Dehydrogenase enzyme (LDH). Neutron Spin Echo spectroscopy and Molecular Dynamics simulations were used to uncover the characteristic length- and timescales of the LDH nanoscale motions in the apo state. The modes involving the catalytic loop and the mobile region around the binding site are activated at room temperature, and match the allosteric reorganisation of bacterial LDHs. In a temperature window of about 15 degrees, these modes render the protein flexible enough and capable of reorganising the active site toward reactive configurations. On the other hand an excess of thermal excitation leads to the distortion of the protein matrix with a possible anti-catalytic effect. Thus, the temperature activates eukaryotic LDHs via the same conformational changes observed in the allosteric bacterial LDHs. Our investigation provides an extended molecular picture of eukaryotic LDH’s conformational landscape that enriches the static view based on crystallographic studies alone.

Protein dynamics and functionality are intimately related. Nevertheless, the fine details of how the conformational changes of proteins modulate and regulate their activity are still to be defined^1^,^2^,^3^,^4^. It is now accepted that protein dynamics is characterized by a hierarchy of timescales, from picoseconds to microseconds, reflecting a rough manifold conformational landscape^5^,^6^,^7^. There have been numerous studies on the relationship between this wide range of dynamical processes and protein functionality. These include substrate binding/unbinding kinetics^8^, catalysis^9^,^10^, and allosteric relaxation^11^,^12^.

To date, experimental techniques such as Nuclear Magnetic Resonance^7^, single molecule spectroscopy^10^,^13^,^14^, time-resolved X-ray crystallography^15^, and Neutron Scattering^16^,^17^ represent the principal means of investigation of protein dynamics and function. Particularly, elastic, quasielastic, and inelastic incoherent NS have been exploited not only to study the sub-nanosecond timescale local functional dynamics of model proteins^18^,^19^ and their solvent^20^,^21^, but also for *in-vivo* investigations of bacterial systems^22^. On the other hand, Neutron Spin Echo spectroscopy (NSE) has been shown to be an invaluable tool to explore the dynamics of biomolecules on larger spatial scales, of the order of nanometer, for times up to hundreds of nanoseconds^23^,^24^,^25^,^26^,^27^. NSE has been successfully applied to systems that exhibit long-range signaling modes via domain displacement, as in the case of the NHERF1^25^, Taq polymerase^23^, and Phosphoglycerate Kinase^26^, as well as to Alcohol Dehydrogenase, a more compact multimeric protein^24^. Because the investigated modes involve length scales that match the size of the proteins, their dynamics overlap with the rigid body motions. Therefore, molecular modelling, e.g. Normal Mode (NM) analysis and Molecular Dynamics simulations (MD), are a necessary tool to disentangle the contribution of specific internal modes, and complement experiments.

In this work we have combined NSE spectroscopy and MD simulations to investigate the thermal activation of the nanoscale motions in a tetrameric protein, the Lactate Dehydrogenase from rabbit muscle 5 (M5) in its apo state.

Because of their dynamical properties and allosteric behaviour, Lactate Dehydrogenase (EC 1.1.1.27) (LDH) is an appropriate enzyme model to decipher the motions involved in the conformational changes that regulate enzyme catalytic activity^28^,^29^. Recent studies have also shown that targeting eukaryotic LDHs with inhibitors, is an efficient way to treat epilepsy and cancers^30^,^31^. Lactate Dehydrogenase is a tetrameric enzyme found in both bacterial and eukaryotic cells, where it catalyses the reduction of pyruvate to lactate using NADH as a coenzyme. Most bacterial LDHs are allosterically regulated showing both homotropic (induced by pyruvate) and heterotropic (induced by fructose 1, 6-bisphosphate, FBP) activations. On the contrary, eukaryotic vertebrate LDHs are considered non-allosteric enzymes^32^,^33^.

Bacterial LDH’s behavior fit the recent unifying models of allostery in which the active (R) and inactive (T) enzyme states coexist in a preexisting equilibrium independently of FBP or substrate binding^4^,^34^. By comparing the crystallographic structures of the bacterial LDHs in apo (T) and holo (R) states, the reorganizations of key parts of the protein matrix upon substrate or FBP binding were identified^35^,^36^,^37^,^38^,^39^. The structural changes of the tetramer include movements of various amplitudes, e.g. the closure of the active site loop, the rearrangement of several mobile regions (MR), and the favorable positioning of catalytic residues. Crystallographic structures of apo/holo eukaryotic LDHs do not show similar conformational changes, suggesting that the protein R state is the preferential one^40^. Moreover it was shown that the active-site loop gating is the rate-limiting step for the catalysis^41^, while its fluctuations present a source of kinetic heterogeneity^42^,^43^,^44^,^45^,^46^,^47^. Seminal study on LDH M5^48^ showed the enzyme’s activation by the presence of pyruvate, with enhanced activity measured upon a thermal treatment. Conformational changes accompanying the enzymatic turnover are thus expected to occur as in the case of the majority of the bacterial LDHs that are allosteric in a proper sense^49^. The presence of long-range correlated motions across the tetramer, potentially relevant for functionality, makes the NSE a pertinent choice to investigate the system. Moreover, the protein crystallographic structure has been solved, offering a good starting point to carry out MD simulations, enabling us to span the protein motion at the atomistic resolution.

We have probed the thermal activation of LDH M5 functional modes and individuated the temperature window where the acquired flexibility favors the sampling of reactive configurations without corrupting the overall catalytic site organization. The localization of the thermally activated modes on the basis of atomistic MD simulations, namely the catalytic loops and the binding site peripheral helices, substantially matches the regions affected by the allosteric reorganization in bacterial LDHs^35^,^36^,^37^,^38^. Thus, at its working temperatures, the LDH M5 in the apo state samples a set of conformations corresponding to the reactive organization of the catalytic site in the holo state. This result shows that temperature regulates functionality in eukaryotic LDHs in a similar way as the allosteric cofactor FBP does for bacterial LDHs. Furthermore, our results show the power of combining NSE and MD simulations to investigate the thermal perturbation of functional flexibility at the nanoscales, therefore breaking ground to a systematic comparison of allosteric proteins optimally working at very different thermodynamic conditions.

## Results

### Protein Diffusion

In this section we present the results on the effective protein diffusion as obtained by NSE spectroscopy. The short time decay of the *I(Q, t*)/*I(Q*, 0) can be approximated by the following cumulant expansion:


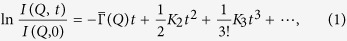


where *K*_2_ and *K*_3_ are the second and third cumulant, and 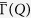 is related to the effective diffusion coefficient *D*_*eff*_ (*Q*) through the relation:


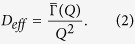


**Figure 1:**
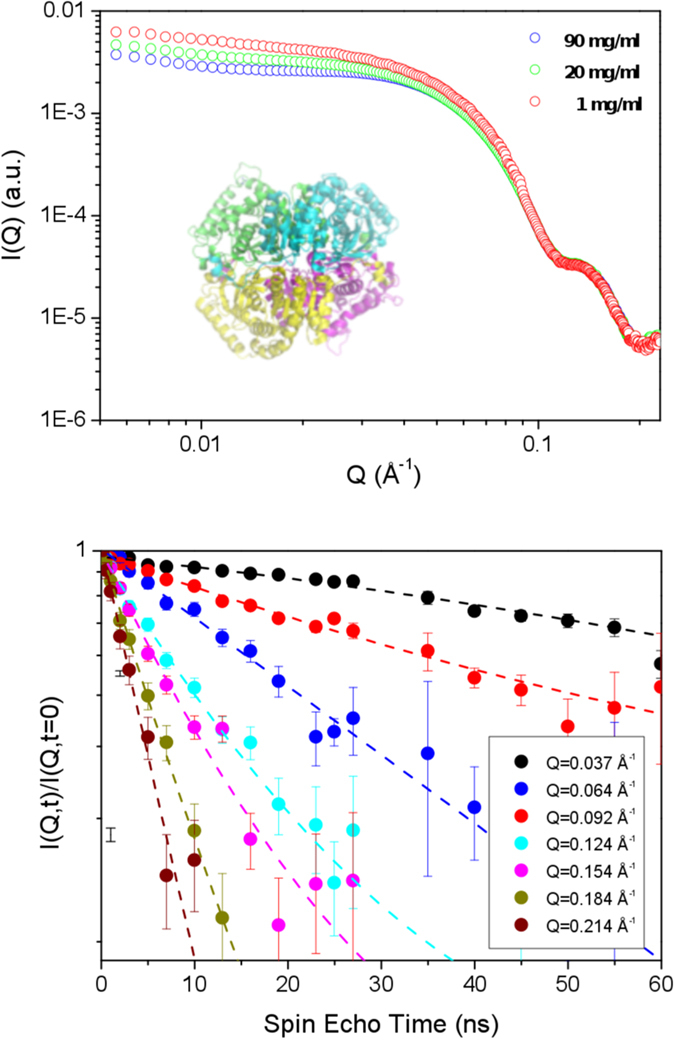
SANS and NSE scattering curves. The upper panel shows the SAXS scattering curves as a function of Q, rescaled to the protein concentrations, and a graphical representation of the protein structure in the inset, where different colors correspond to different subunits.The lower panel shows the intermediate scattering function *I(Q, t*)/*I(Q*, 0) as a function of the spin-echo time (circles) measured at T = 298 K, shown for different *Q* along with the fits to the data (lines).

The *Q*-dependent diffusion of the protein can therefore be extracted by fitting the normalized intermediate scattering function *I(Q, t*)/*I(Q*, 0), as shown in Fig. 1 for data at T = 298 K and for representative values of *Q*. The lines correspond to the least square fit performed over the time window 0–50 ns of the *I(Q, t*)/*I(Q*, 0) data obtained at T = 298 K. The data have been described by the first two terms of the cumulant analysis at all temperatures.

Since we measured protein solutions at a concentration where protein-protein interactions may be significant, we calculated the single-protein diffusion coefficient in the infinite dilution limit *D*_0_(*Q*) from the effective diffusion *D*_*eff*_ (*Q*) of Eq. 2, by considering concentration-dependent inter-protein interactions and solvent-mediated interactions^50^:


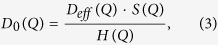


where *H(Q*) is the hydrodynamic function representing *Q*-dependent hydrodynamic interparticle interactions mediated by the solvent, while the structure factor *S(Q*) describes the direct inter-protein interactions.

The *S(Q*) was obtained, as described in the methods section, from the extrapolation of SAXS curves at infinite dilution using Eq. 5. The background-corrected SAXS curves are displayed for different protein concentrations in the upper panel of Fig. 1, after rescaling by the concentration C (mg/ml). As expected, at high *Q*, the scattering curves are unaffected by the inter-protein interactions and overlap. The temperature effect on the *S(Q*) was estimated by performing SAXS measurements at different temperatures, showing only the low *Q*-region to be affected.

**Figure 2:**
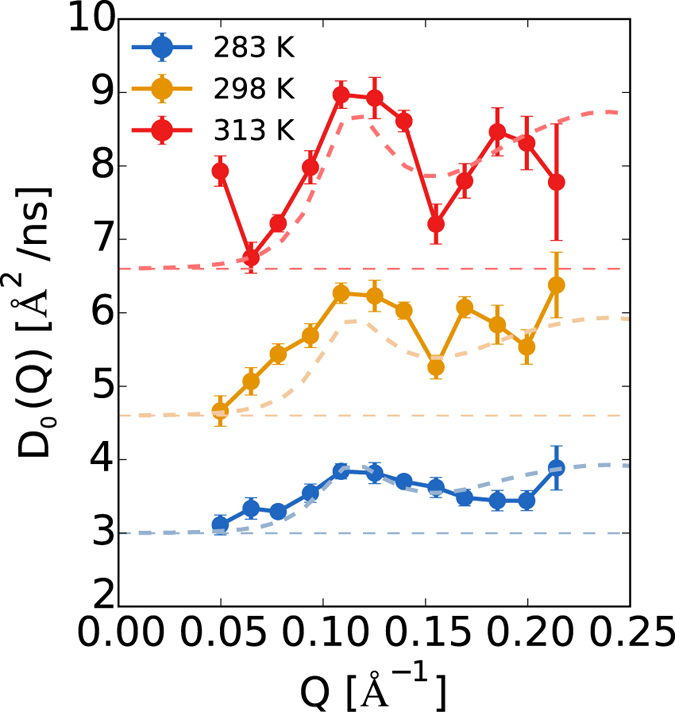
Diffusion spectra at three different temperatures. Circles indicate the experimental points, the horizontal dashed lines indicate the value of the translational diffusion evaluated by DLS measurements. The dashed lines curves indicate the *Q*-dependent diffusion coefficient calculated for a rigid-body (X-ray structure) and using the mobility tensors *D*^*R*^ and *D*^*T*^ calculated by the HYDROPRO program^52^.

Following the procedure described in ref. 24, to account for the hydrodynamic contribution, we have scaled the term *D*_*eff*_(*Q*) · *S(Q*) to match the *Q*-independent value of the translational diffusion measured by DLS. Theoretical calculations on model solutions of spherical charged proteins have shown *H(Q*) to have a similar trend to *S(Q*) and, quite importantly, that it does not manifest significant oscillatory behavior in the *Q*-range above 0.06 Å^−1 ^51. Thus, any modulation of the *D*_0_(*Q*) spectrum, reported in Fig. 2, can be ascribed to the single tetramer dynamics.

At all temperatures, the spectra show a well-defined first peak at Q = 0.11 Å^−1^, whose intensity grows with temperature. This peak, at the characteristic length 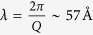, relates to the correlated motion of regions of the tetramer at the external surface. A second peak is visible at shorter length scales, namely for *Q* approaching the value 0.2 Å^−1^. The resolution of the second peak region is somewhat poorer thus we concentrate our efforts in discussing the first peak only. In Fig. 2 we also reported the theoretical curves for the rigid body roto-translational diffusion calculated according to ref. 23:


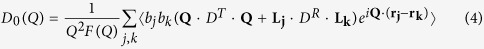


where *D*^*T*^ and *D*^*R*^ are the translational and rotational diffusion tensors, respectively, the sum runs over the atoms of the proteins, *b*_*k(j*)_ indicates the scattering lengths for the scattering center *k(j*), **L**_*k(j*)_ = **r**_*k(j*)_ × **Q** is its angular momentum vector, and *F(Q*) is the form factor for the protein. The tensors *D*^*T*^ and *D*^*R*^ were obtained by hydrodynamic calculations performed using the HYDROPRO software^52^. The values for the translation diffusion coefficients obtained by DLS (horizontal baselines in Fig. 2) and those obtained by HYDROPRO are close at all considered temperatures, they differ by a factor 1.2 (see SI Table S1). This factor was used to rescale the elements of the translational and rotational diffusion tensors *D*^*T*^ and *D*^*R*^ in Eq. 7 for a correct comparison with the experimental spectra. The plotted curves immediately reveal that the rigid body rotational diffusion (dashed curves) accounts for the main features of the spectra, apart from the peak zone around 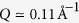, which contains an additional contribution at 298 K and 313 K. The latter stems from the internal motion of the protein that, along with the rotation and the translation, contributes to the the overall protein diffusivity: 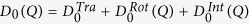. The internal contribution is negligible at the lowest temperature T = 283 K but, at higher temperatures, it represents 10–15% of the total diffusion of the protein when translation is removed.

The contribution from the internal *Q*-dependent motion can be dissected by using NM analysis^53^ and focusing on the low frequency modes. These have been extracted for the crystallographic configuration, see SI Figs S1 and S2. Because of the highly symmetric nature of the LDH protein, at variance with previously investigated systems where large domains displacements occur at low frequency^23^,^25^, it is difficult to single out a dominant mode. Therefore, in order to gather microscopic insights on the protein internal dynamics, we have performed long MD simulations and estimated the contribution of internal motion to the *D*_0_(*Q*) spectra.

### Protein Internal Motion

Following the work flow proposed by Smolin *et al*.^54^, the diffusion spectrum *D*_0_(*Q*) extracted from the MD simulations can be decomposed in its translational, rotational, and internal contributions by adequate post-processing of the trajectories. For instance, by removing the translation of the protein’s center of mass from the original MD trajectory and fitting on it a rigid reference structure, a virtual trajectory is generated where only the rigid body rotation is present. In the same spirit, if the MD trajectory is fitted, frame by frame, on a reference structure, thus removing roto-translation, only the internal modes will be maintained. The processed trajectories are used in the calculations of the intermediate scattering function *I(Q, t*) in order to extract respectively the rotational diffusion 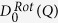 and the internal diffusion 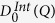 by fitting the initial decay of the obtained *I(Q, t*)/*I(Q*, 0). However, when employing this strategy for a direct comparison with experimental data, *ad hoc* numerical manipulations are needed. In fact, molecular force-fields routinely used in MD simulations of protein-water solutions generally bias protein motion and empirical rescaling of roto-translation is necessary^54^. Moreover, care should also be placed on practical issues such as the system-size dependency as well as the accuracy of the fitting procedure to extract the diffusion coefficients^55^.

**Figure 3:**
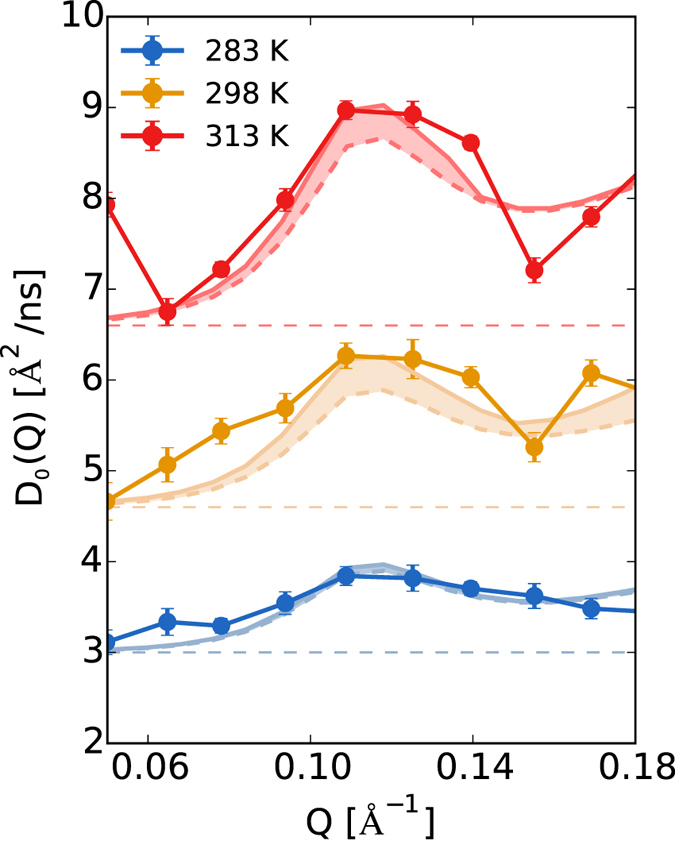
Internal contribution to diffusion spectra. Experimental diffusion spectra at different temperatures compared to the theoretically reconstructed spectrum (solid curve) obtained by adding the rigid-body contribution (dashed curve) to the internal-dynamics contribution derived from long MD simulations (shown in the shaded area).

In order to limit the impact of these weaknesses, we used MD-based calculations for the internal motion only. From our MD simulations, we removed roto-translation, and obtained the internal component of the spectra. The internal diffusion has been added to the curve calculated by considering the rigid-body motion as described in the previous section and compared to the experimental data, see Fig. 3. Most notably, we observe the activation of the internal motion at T = 298 K. Between T = 298 K and T = 313 K, the internal contribution at the peak is comparable (0.4 Å^2^/ns), becoming much larger at highest simulated temperature, T = 330 K (~0.8 Å^2^/ns), see Fig. S3 in SI. Importantly, the addition of the internal contribution allows to quantitatively reproduce the experimental value of the diffusion coefficient *D*_0_(*Q*).

**Figure 4:**
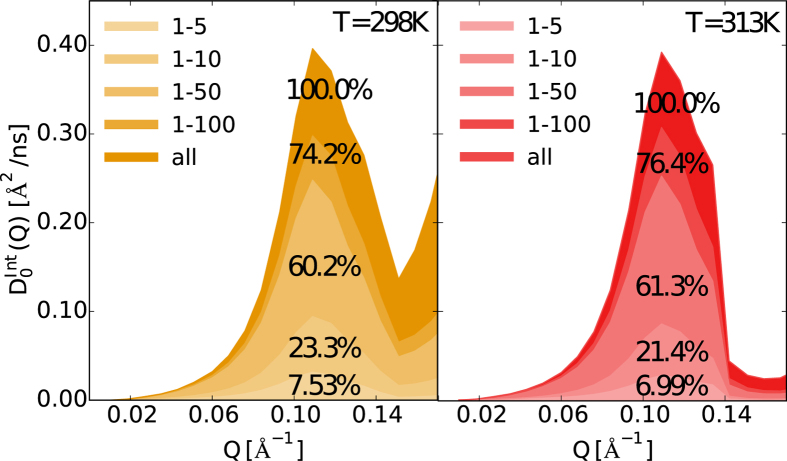
Contribution of the principal modes to the diffusion spectra at temperature 298 K and 313 K.

To inspect these thermally activated internal protein modes in a length-range corresponding to the first peak of the calculated spectra, and to further understand the temperature effect on these modes, we performed Principal Component Analysis (PCA)^56^ on the processed trajectories with removed roto-translations, at T = 298 K and T = 313 K (see section Methods). By projecting these trajectories on a different number of modes, we are able to extract the contribution of specific internal motions to *D*_0_(*Q*). This is done by calculating, and subsequent fitting, *I(Q, t*)/*I(Q*, 0) from the projected trajectories. For the thermally activated state (T > 283 K), it is found that the first 100 modes account for ~75% of the diffusive contribution in the first peak, see Fig. 4 and Table S2 in SI. The regions of the protein interested by these modes are highlighted in Fig. S4 in the SI, clearly showing that the larger flexibility induced by the modes localizes at level of the loops on the protein surface, most notably the catalytic site loop, and that this distribution of flexibility is the same for the four subunits.

**Figure 5:**
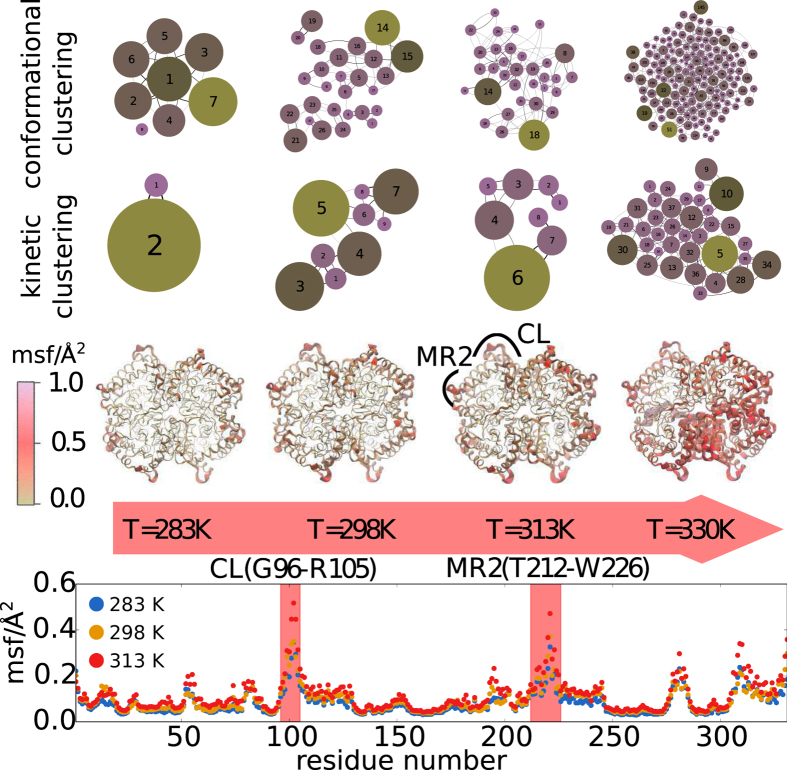
Network of conformational states visited by the LDH protein in MD simulations at different temperatures. In the top layers we report the networks obtained by conformational and kinetic clustering, respectively. Each node of the network represents a conformational substate, the size of the node is proportional to its occupancy. The color scale in the network is used to further stress different occupancy of the conformational states. In the bottom layers, the flexible regions of the protein individuated by the local atomistic fluctuations are highlighted in the protein structure and along the subunit sequence. We have specifically indicated the loop of the catalytic site (CL) and the adjacent helical region (MR2 according to the annotation of ref. 36).

We now provide a complementary view of the protein nanoscale modes underlying the internal motion by performing conformational and kinetic clustering of the MD trajectories, see section Methods. The results are reported in the Fig. 5. In the upper layer of the graph, the network of states visited during the dynamics is represented for the four simulated temperatures. At the lowest temperature, only few conformational states are accessed by the protein at the 0.6 *μs* time scale, this number increasing exponentially with temperature (see Table S3 in SI). While conformational clustering classifies conformational states only on the basis of their proximity according to the RMSD, a more subtle casting is achieved by merging together the frequently interconverted states, yielding a representation of states that are mutually distinguished by high kinetic barriers, as represented in the middle part of the Fig. 5 57,58. The thermally activated conformational disorder is in fact due to different orientation of the binding site loops and adjacent peripheral helices, as the reader can appreciate from the third layer of the Fig. 5, where the protein regions manifesting the highest flexibility are magnified in proportion and accentuated in color. The pictorial representation of the protein is complemented by the sequence profile of the mean square fluctuations for one subunit in the last layer. Among the flexible regions activated in temperature, we individuated two of the principal protein regions involved in the allosteric reorganization of bacterial LDHs^35^,^36^,^37^,^38^; the active site loop and region MR2 around the E222 residue.

The results of the PCA and the clustering clearly show that the contribution to internal diffusivity stems from an ensemble of modes involving the peripheral regions nearby the catalytic site. This collective reorganization of the protein matrix is the source of the plasticity necessary for functionality, i.e. the conformational shifts due to cofactor and substrate binding, as well as the gating of the binding site loop required for efficient catalysis. Since the binding site loops play a fundamental role in LDH activity^41^,^42^,^43^,^44^, we have specifically targeted them in further investigation by considering their correlated motions. The distances between the loops span the range 36–75 Å (see Fig. S6 in SI), their correlated motions therefore falling in the probed region of the peak, 0.08 Å^−1^ < Q < 0.14 Å^−1^. By using the harmonic approximation, details being provided in the Methods section, it is found that specific correlated motions account for about 5–15% of the internal diffusivity in the peak region, see Table S4 in SI. The same is found by processing a trajectory containing the internal dynamics only, and excising the loop region, see Fig. S5 in SI. Interestingly, the thermal response of the correlated loop motions seems to depend on the considered subunit, see Figs S6, S7, and S8 in SI. The obtained results reinforce the notion that a wide set of correlated motions across the four subunits contribute to the internal component of the diffusivity peak. It is important to note that the relative distances across the four subunits between the flexible amino acids in the MR2 stretches are in the range of ~36 Å, probably contributing to the high *Q*-region of the spectra, while the distances across the four subunits between MR2 and the active site loop atoms amount to 60 Å for adjacent and intra-dimer subunits, and 80 Å for diagonal distances, respectively. Thus the correlated fluctuations of the catalytic loop with the MR2 region are a likely contributor to the observed first peak in the diffusion spectrum.

## Discussion

NS spin-echo spectroscopy enabled us to probe the thermal activation of the large-scale motions in a mesophilic tetrameric LDH enzyme, relevant for its activity. At the lowest investigated temperature, T = 283 K, the protein motion is substantially dominated by rigid body roto-translation. Only from the ambient temperature condition do the internal motions give a significant contribution to the *Q*-dependent diffusivity. At *Q* ~ 0.11 Å^−1^, this contribution *D*^*Int*^ is about 0.4 Å^2^/ns, and is equal at 298 K and 313 K. This finding suggests that in the optimal temperature window for the protein activity, 298 K < T < 313 K^59^, protein requires and sustains a steady level of internal flexibility. Additional thermal excitation would have a degrading effect on activity by provoking the distortion of the catalytic site and dissipating conformational changes in non-functional paths. LDH maximal activity occurs at ~313 K and is compromised at higher temperatures^59^. The loss of activity anticipates the thermodynamic unfolding detected at 330–340 K^60^,^61^. On the basis of MD simulation in this high temperature regime, our findings identify that high flexibility interests a greater portion of the protein and induces a substantial distortion of the structure around the catalytic pocket.

The analysis of the MD trajectories based on PCA and clustering strategies highlight that the functional internal diffusivity cannot be reduced to a single dominant motion, but is rather attributed to a wide range of modes covering the peripheral region of the protein around the catalytic sites, the binding site loops Ala95-Arg105 (CL), and the helices Arg105-Ser127 (MR2 region). It is speculated that the thermal activation of some of these modes are fundamental to the functionality of the protein similarly to the allosteric regulation in bacterial LDHs. In fact, by comparing the X-ray structures of apo and holo bacterial LDHs^36^,^37^, it was possible to single out the regions involved in the allosteric conformational shifts.

In our experiments and simulations, the protein is in the apo state, where we always find the binding site loop sampling the open configuration. The closed loop configuration, which is rate-limiting the catalysis, is most likely only accessible upon co-factor and substrate binding. However it is important to note that in the MD simulations, we observe the reorganization of the catalytic site toward a reactive configuration as the temperature increases. This is in agreement with the experimental observation that a heat shock treatment of rabbit M5 LDH^48^ increases the enzymatic efficiency. In bacterial allosteric LDH, the thermal energy has shown to strongly favor the active (R) state population by dramatically increasing the affinity for pyruvate even in the absence of FBP^38^.

**Figure 6:**
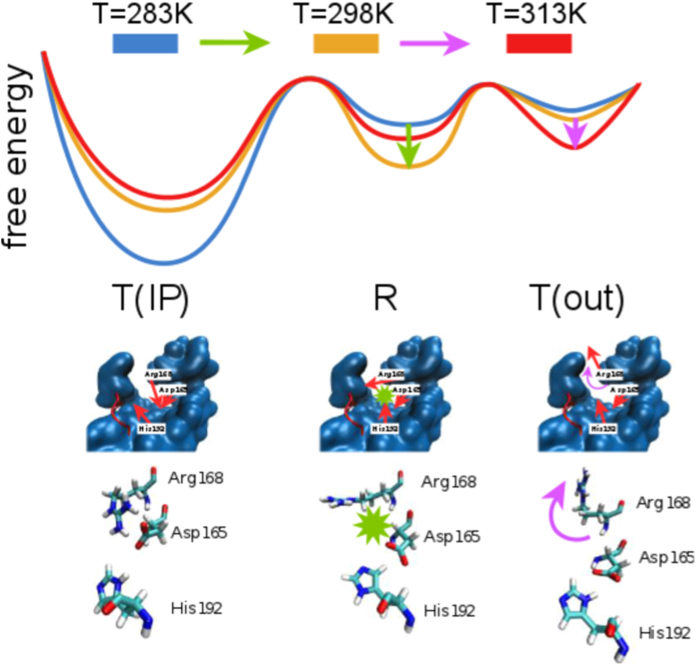
Schematic representation of changes in relative populations of states sampled in the simulations: T(IP) - inactive (T) state with ion pair (IP) formed between Arg168 and Asp165, R - active state, T(out) - inactive state where Arg168 points outside of the active site (out). Increasing the temperature to T = 298 K allows for sampling of the active state (R) by breaking the Arg168-Asp165 ion pair (marked by a green star and arrow) and allowing the Asp165 and His192 to interact. Further increase in temperature deactivates the enzyme (T), and causes the rotation of the Arg168 sidechain outside of the active site (out), shown by a purple arrow. The portion of the protein shown in blue is surrounding the active site, while the catalytic loop is shown in red. The red arrows show the side chain orientation in the middle panel. The lowest panel shows the three amino acids from the simulation snapshots.

At variance with bacterial LDHs, in the rabbit muscle 5 LDH, the extension of a supplemental N-ter helix from one domain to another locks the movement of the MR1 region (Leu164-Gly186), the latter found to be quite rigid in our structure. In bacterial LDHs, the displacement of MR1 from the apo (T-inactive) to the holo (R-active) state allows the reorganization of the active site, most notably by inducing the correct positioning of the substrate binding residue, equivalent to Arg168 in the rabbit muscle 5 LDH, toward the oxamate. In our system, the positioning of Arg168 toward the reactive configuration is enhanced by the temperature increase, see Fig. 6. This is shown by monitoring the conformational fluctuations of the residue side chain that oscillates between an extended configuration, mimicking the configuration assumed when pyruvate is bound in the catalytic pocket, to a state bound to the Asp165. Our results challenge the static view based on the comparison of crystallographic structures that show the eukaryotic LDHs only in the R-active state. We found a salt-bridge interaction between Arg168 and Asp165 in the rabbit LDH molecules, pointing to the existence of a new representative of the T-inactive state, different from the one observed in bacterial LDHs.

Between 298 and 313 K, the temperature eases the breaking of the ion pair Asp165-Arg168 and increasingly favors the extension of Arg168 as well the interaction of Asp165 with the catalytic His192, see SI. Further increasing the temperature, the Arg168 starts visiting conformations pointing outside the active site, which resemble the inactive (T) state of bacterial LDHs. For a complete overview of the process, see Fig. 6 and the discussion of Figs S9 and S10 in SI. The analysis of the conformational shifts induced by the coenzyme and substrate binding^24^,^58^,^61^ will be the next step of the investigation.

In conclusion, by combining NSE spectroscopy and MD simulations, we have shown that temperature induces functional conformational changes in the eukaryotic M5 LDH that closely match the allosteric reorganisations detected in bacterial LDHs. The experimental and numerical tools presented here allow the characterisation of the multiple time- and length scales of protein dynamics with a specific focus on functionally relevant modes. The exploration of the thermal response of these modes is essential in comparing proteins with different optimal working temperatures^62^ and addressing different evolutionary mechanisms of functional regulation.

## Methods

### Protein preparation

Rabbit muscle lactic dehydrogenase was purchased from Sigma in the form of an ammonium sulfate crystalline suspension. A 200 ml sample containing 2000 mg of enzyme was extensively dialyzed against a buffer containing 50 mM Tris-HCl pH 7.5 and 25 mM NaCl. The solution was then concentrated 10 times using Amicon-ultra concentrators with 30 kDa cutoff (Millipore). In order to eliminate the small amount of contaminants, the concentrated fraction was divided in small aliquots of 500 μl which were injected on a superpose 12 column (GE Healthcare Life Sciences) equilibrated with 50 mM NaCl buffered with 50 mM Tris-HCl pH 7.5. 40 fractions of 0.3 ml were collected using a FPLC chromatographic system (GE Healthcare Life Sciences). The most concentrated fractions of the eluted peak were pooled. These fractions were concentrated and dialyzed several times against a solution of pure deuterium oxide (Sigma-Aldrich) buffered with 100 mM MES pH 5.8, 50 mM NaCl. Prior to experiments, the samples were centrifuged at 10000 g to remove small aggregates. The final samples contained 4 ml of rabbit LDH muscle 5 at 90 mg/ml with a D_2_O concentration of the buffer higher than 98%.

### Neutron Spin Echo

One of the most prominent features of Neutron Scattering is the ability to relate dynamical processes to the length-scales at which they occur. This feature, and the fact that neutrons interact with the matter causing virtually no radiation damage, makes NS a very effective approach to probe complex multi-scale dynamical processes in biological samples. In most common NS spectroscopy experiments, one measures the so-called dynamic structure factor *S(Q, E*), where the characteristic sub-nanometer and nanosecond length- and time-scales of biomolecule motions can be derived from respectively the wavevector-transfer *Q* and energy-transfer *E* dependence. On the other hand, neutron spin echo (NSE) spectroscopy returns the Fourier transform of the dynamic structure factor, yielding the intermediate scattering function *I(Q, t*)/*I(Q*, 0) directly in the time domain. By encoding the velocities of polarized neutron in their precession motion across a highly homogeneous magnetic field^63^, NSE techniques extends the accessible time domain up to hundreds of nanoseconds.

In simple words, NSE allows to follow the time evolution of the correlated and diffusive motion of protein atoms at the nanosecond timescale. In a typical NSE experiment on protein systems, the characteristic length-scales that can be explored, by acting on the scattering vector *Q*, are of nanometer order. At these characteristic lengths the signal mainly comes from the correlated motion of separated atoms (coherent contribution). Therefore, the NSE is a very pertinent technique to sample, at a given temperature, the relative motions of protein domains that are typical of large-scale conformational changes.

The experiments were performed on the J-NSE spectrometer at the FRM-II reactor in Munich at two wavelengths, 8 and 10 Å, giving a maximum spin echo time of 65 ns in a *Q*-range 0.037 < *Q* < 0.214 Å^−1^. The protein concentration in the buffer was 90 mg/ml, well higher than that corresponding to the dilute regime, necessary to achieve a sufficient spin echo signal. Both the protein solution and the buffer alone were measured at the same experimental conditions to allow background subtraction. The samples were investigated at 3 temperatures: 283 K, 298 K, 313 K.

### Small Angle X-Ray Scattering

Small-Angle X-ray Scattering experiments were performed at the high-brilliance beamline ID02 at the European Synchrotron Radiation Facility (ESRF) in Grenoble, France. A sample to detector distance of 2 m was chosen to cover a *Q*-range 0.05 ≤ Q ≤ 3 nm^−1^. The incident X-ray wavelength *λ* was 0.1 nm. The measurements were performed in a Peltier-controlled flow-through capillary of 1.8 mm diameter to minimize beam damage of the samples and to ensure an accurate subtraction of the background (buffer solution). The two-dimensional scattering patterns were recorded using a Rayonix MX-170HS fiber-optic taper coupled CCD camera. The two-dimensional spectra were normalized to absolute intensity scale after applying the detector corrections for spatial homogeneity and linearity. Normalized SAXS patterns were azimuthally averaged to obtain the one-dimensional scattering profiles [*I(Q*) vs. *Q*].

The scattering *I(Q*) of a solution of *N* proteins is proportional to the product of the structure factor *S(Q*), associated to the concentration-dependent interaction between different proteins, and the form factor *F(Q*), which accounts for the spatial correlations of the atoms within the single protein and is independent of the concentration, *I(Q*) ~ *NS(Q)F(Q*). At low concentration, the interaction is negligible (*S(Q*) ~ 1), so that one can estimate *F(Q*) directly by extrapolating the concentration-dependent scattering to *C* = 0. On the other hand, at higher concentrations, a change in the scattering intensity at low *Q* is caused by the inter-particle interactions, i.e. the *S(Q*) can be expressed as:


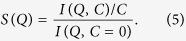


The samples were investigated by SAXS at the same temperatures mentioned in the previous section. It is worth nothing that the optimal way to obtain *S(Q*) would have been by using Small-Angle Neutron Scattering that gives the same scattering density distributions of NSE. However, the S(Q) deviates from unity only at low Q, i.e. in a low spatial resolution regime, where the precise details of the atomic density distribution are negligible. In this regime the two techniques return equivalent results for our purpose.

### Dynamic Light Scattering

DLS measurements were carried out using an ALV CGS-3 Compact Goniometer equipped with a HeNe Laser with a wavelength of 632.8 nm, a 22 mW output power, and an ALV LSE-5004 Correlator. Samples were measured at a scattering angle of 90°, while the sample temperature was controlled via an external water bath circulator.

### Molecular Dynamics Simulations

The protein structure, as obtained by X-ray scattering (PDB 3H3F, chains E, F, G, H), was embedded in a simulation box containing 34,275 water molecules, producing a system of 123,627 particles including counter-ions. We used the NAMD 2.9 software^64^ and the CHARMM22/CMAP force-field^65^ to perform all-atom simulations in the NPT ensemble and using periodic boundary conditions. Water was modelled by the TIP3/CHARMM model^66^. Four simulations were carried out at P = 1 atm and T = [283 K, 298 K, 313 K, 330 K], where the first three temperatures match those in experiments, and the fourth further probes the high-temperature regime. The integration time step was set to 2 fs. The non-bonded interactions and the short range electrostatic interactions were cut off at 9 Å, while the long range electrostatic interactions were treated with the PME algorithm with a grid spacing of 1.3 Å. After initial equilibration, the simulations were run another 0.6 *μ*s, and the latter were used for analysis purposes. The trajectories were recorded with a frequency of 5 ps.

### Analysis of Molecular Dynamics Trajectories

#### Conformational and Kinetic Clustering

As a probe in exploring the change in protein flexibility as a function of temperature, conformational clustering was employed. This technique reduces the large number of conformational substates visited by the protein during a simulation to a subset of representative states, the cluster leaders, and keeps track of their interconversion^57^. In this work, we used the root mean square displacement (RMSD) among conformations as an order parameter to distinguish the different substates with a cut-off of 1.5 Å. From the operational point of view, the protein conformation visited at time *t* is compared with the structures of the leaders individuated for *t*′ < *t* by computing:


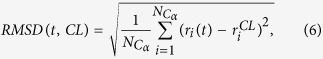


where the sum runs over the number 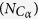 of *C*_*α*_ in the protein, and index *CL* indicates the structure of a cluster leader. Clustering is performed by the leader algorithm^67^. The conformational network is used as a starting point for a subsequent clustering operation, the Markov clustering^68^, so as to obtain kinetically separated states, see for instance refs 57,58. The frequencies of interconversions between the conformational cluster leaders allow to build a transition matrix, which is used to run random walks on the networks and individuate the states where the walkers are kinetically trapped.

The networks of substates obtained by both strategies and their interconversions are graphically visualized by using a force-based algorithm implemented in GEPHI^69^.

#### Principal Component Analysis

To decompose the complex dynamics of the multimeric LDH to independent motions, we used Principal Component Analysis (PCA)^56^ available through the GROMACS package^70^. By using the PCA, the principal modes on which the larger fluctuations of the protein motion is concentrated can be individuated and additionally, a virtual trajectory projected on these modes can be obtained in order to quantify the contribution of these modes to the Neutron Scattering spectra.

#### Diffusion in Harmonic Approximation

In order to characterize modes relevant for functionality, we estimated the diffusivity associated to particular elements of the LDH, namely for the distance between each subunit pair (*α, β*) of the catalytic loops (Ala95-Arg105). The collective variable (CV) combining the distances between the *C*_*α*_ atoms in the loops is first designed:


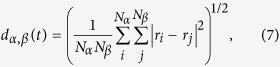


with the sum running over the *C*_*α*_ atoms in the loops *α* and *β*. The diffusion of the relative collective distance between the loops, fluctuating around their equilibrium values, is estimated in the harmonic approximation^71^ as 
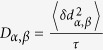
. The term 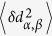 is the mean square fluctuations of the variable and *τ* its decorrelation time, extracted from the time correlation function 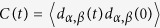 by using an exponential fit for the initial decay.

## Additional Information

**How to cite this article:** Katava, M. *et al*. Thermal activation of ‘allosteric-like’ large-scale motions in a eukaryotic Lactate Dehydrogenase. *Sci. Rep.* **7**, 41092; doi: 10.1038/srep41092 (2017).

**Publisher's note:** Springer Nature remains neutral with regard to jurisdictional claims in published maps and institutional affiliations.

## Supplementary Material

Supplementary Information
