# Supplementary material for: Thermal activation of ‘allosteric-like’ large-scale motions in a eukaryotic Lactate Dehydrogenase
Source: Sci Rep. 2017 Jan 23;7:41092. doi: 10.1038/srep41092 (PMC5253740; doi:10.1038/srep41092)
Supplement: Supplementary Information [file srep41092-s1.pdf]

Supplemental Information: Thermal activation of 'allosteric-like' large-scale motions in a eukaryotic Lactate Dehydrogenase.

Marina Katava[a], Marco Maccarini[b,1], Guillaume Villain[a], Alessandro Paciaroni[c], Michael Sztucki[d], Oxana Ivanova[e], Dominique Madern[f,1], and Fabio Sterpone[a,1]

[a]Laboratoire de Biochimie Théorique, IBPC, CNRS UPR9080, Univ. Paris Diderot, Sorbonne Paris Cité, 13 rue Pierre et Marie Curie, 75005, Paris, France
[b]Univ. Grenoble Alpes - Laboratoire TIMC/IMAG UMR CNRS 5525, Grenoble Pavillon Taillefer Domaine de la merci, 38700 La Tronche,France
[c]Dipartimento di Fisica e Geologia, Universitá di Perugia, via A. Pascoli, 06123 Perugia, Italy
[d]European Syncrotron Radiation Facility, 6, rue Jules Horowitz, 38042, Grenoble, France
[e]Jülich Centre for Neutron Science (JCNS) at Heinz Maier-Leibnitz Zentrum (MLZ), Forschungszentrum Jülich GmbH, Garching, Germany
[f]Institut de Biologie Structurale (IBS), Univ. Grenoble Alpes, CEA, CNRS, 38044 Grenoble, France
[a]Laboratoire de Biochimie Théorique, IBPC, CNRS UPR9080, Univ. Paris Diderot, Sorbonne Paris Cité, 13 rue Pierre et Marie Curie, 75005, Paris, France
[1]To whom correspondence should be addressed. E-mail: marco.maccarini@imag.fr, dominique.madern@ibs.fr, fabio.sterpone@ibpc.fr

Translational Diffusion

The translational diffusion measured by Dynamic Light Scattering experiment is compared to the theoretical estimate based on hydrodynamic calculations[1] (software HYDROPRO) on the crystallographic structure of the proteins. The theoretical estimate at all temperatures is about a factor 1.2 larger than the experimental data. The data are reported in Table S1.

Normal Mode Analysis

In Figure S1 we report the diffusion coefficient $D_0^{Int}(Q)$ obtained from projecting the normal modes of the X-ray structure PDB 3H3F on vectors scattered over a sphere so as to mimic a point scatterer. For each mode α, the diffusion coefficient is calculated as follows: $D^\alpha(Q) = \frac{C}{Q^2 F(Q)} \langle \sum_{k,l} b_k b_l \exp[i\mathbf{Q}(\mathbf{r}_k - \mathbf{r}_l)](\mathbf{Q}\cdot\mathbf{e}_k^\alpha)(\mathbf{Q}\cdot\mathbf{e}_l^\alpha)\rangle$, where $F(Q) = \sum_{k,l} b_k b_l \exp[i\mathbf{Q}(\mathbf{r}_k - \mathbf{r}_l)]$, and $C = \lambda_\alpha \frac{k_B T}{m\omega_\alpha^2}$, where ω_α^2 is the eigenvalue associated to each mode, and λ_α is the mode-dependent relaxation rate, containing friction coefficients within the molecule and with the surrounding water. We have not estimated the prefactor C and thus D is reported in arbitrary units. Note that mode 7 and 9 overlap in the Figure S1. In Figure S2 the first 6 nontrivial Normal Modes of the X-ray structure PBD 3H3F are pictorially represented. The magnitude and the direction of the arrows reflect the intensity and the direction of movement in the harmonic perturbation.

Internal Contribution to Diffusivity Spectrum

In Figure S3 we report internal dynamics contribution D_0^{int} to the Q-dependent diffusion coefficient as obtained from post-processing of the MD trajectories (removing roto-translational rigid-body motions) and by fitting the calculated $I(Q,t)/I(Q,0)$. The curves refer to the different temperatures used in the simulations.

Principal Component Analysis

The Principal Component modes contribution to the internal dynamics at the first peak region ($Q = 0.118$ Å^{-1}) is given in Table S2. The protein trajectory is projected on a different number of Principal Component modes, and the internal contribution to

the Q-dependent diffusion constant is evaluated. The percentage of the total internal dynamics at$Q = 0.118$ Å^{-1} described by these components is reported in parentheses. In Figure S4 we represent the mean squared fluctuations of C_α atoms calculated along the MD trajectory obtained at T=298 K and projected on different sets of Principal Component modes. The protein regions showing larger fluctuations are magnified (in size and color scale) in the molecular representations. The most flexible regions, namely the catalytic loop (CL) and the mobile region (MR2) are indicated by semicircular lines.

Conformational Clustering

The number of conformational states obtained by conformational and kinetic clustering of the MD simulations is reported in Table S3. The conformational clustering was based on the collective variable RMSD and using a cut-off of 1.5 Å. The total number of clusters obtained is indicated in the first column,$N(t_{sim})$. In the third and fourth columns, we report the parameters of a simple exponential growth model used to fit the data, $N(t) = N_\infty \cdot (1 - exp(t/\tau))$. In the last column we report the number of independent kinetic states as obtained by applying Markov state model based clustering algorithm with a threshold of 2.0. At the highest temperature ($T = 330K$) the exponential model does not apply.

Dynamics Excluding Active Site Loops Correlations

The contribution of the correlated motion of the active site loops is analysed considering first a virtual trajectory where the dynamics of the loops was removed ('no loop'). Using this trajectory the internal dynamics diffusion coefficient is extracted from fitting the $I(Q,t)/I(Q,0)$. The results obtained at T=298 K show that the loops account for 9% of the peak. The correlated inter-subunit loop dynamics is thus characterised by a diffusion coefficient of 0.037 Å^{2}ns^{-1} at the peak, which agrees reasonably well with calculations of interloop correlations evaluated in the framework of the harmonic approximation (see Table S4). In the harmonic approximation the diffusivity associated to the interloop separation is obtained as$D_{\alpha,\beta} = \frac{\langle \delta d^2_{\alpha,\beta} \rangle}{\tau}$, where d is the interloop distance defined for all atoms in the loop as $d_{\alpha,\beta} = (\frac{1}{N_\alpha} \frac{1}{N_\beta} \sum_i^{N_\alpha} \sum_j^{N_\beta} |r_i - r_j|^2)^{1/2}$, and τ is the relaxation time of the autocorrelation function of this distance. The time evolution of the loop separations and the associated time autocorrelation functions are reported in Figure S6 and S7, respectively. The calculated diffusion constants for loops correlated motions are reported as a function of temperature in Figure S8. The contribution of correlated inter-loops displacements to the diffusivity spectra could be estimated alternatively by performing simulations with loop motions restrained. Note however that in this case the restrains could affect the motion of adjacent regions of the protein and induce some artefacts.

Conformational States of the Catalytic Site

In Figure S9 we report the distance variations in Å between the amino-acids R168 and D165 for each subunit of the apo-state protein and at various temperatures during the 0.6μs MD simulations (labeled E, F, G, and H). The distance of approximately 4 Å corresponds to the R168 lateral chain protruding within the catalytic site and forming a salt bridge with D165, as illustrated by the representative view extracted from the simulation at 283 K in Figure S10. A distance higher than 4 Å, typically between 6 and 8 Å, indicates that the R168-D165 salt bridge is broken, as shown in representative conformation extracted from the MD simulation at 298 K, see Figure S10. The distance of 10 Å indicates the R168 lateral chain is located outside of the catalytic site as it is illustrated in the snapshot of active site of the subunit E taken at the end of the simulation at 313 K, see Figure S10. For sake of comparison, a close-up view of the ternary complex crystal structure (PDB code 3F3H) is shown with the same orientation. The substrate analogue (oxamate) is colored in red, while the coenzyme is shown in orange. Subunits E and F are in green and blue, respectively. For the sake of clarity, other subunits are not shown. Important residues are represented in sticks. It should be noted that the residue D165 helps to polirize the catalytic residue H192 during the catalysis. In fact, this interaction allows the nitrogen (N3) of the histidine ring to be protonated. This positive charge participates in the stabilization of the substrate which is negatively charged during the transition state[2]. Because of the neutral pH used for the simulations, H192 is non-protonated. Simulations that take into account different protonation states of histidine were out of the scope of the study. Possible changes of the protonation state change upon conformational shifts were therefore not considered here. R168 lateral chain is orientated within the catalytic site and interacts with the negatively charged analogue. The R168-D165 distance in the X-ray structure of the holo state is ∼6.5 Å.

The MD simulation data show that in the apo state, as is expected for an eukaryotic LDH, R168 from rabbit LDH is mainly located within the catalytic site at 283 K. Because there is no substrate analogue, R168 forms a strong salt bridge with D165, and fluctuates around this tight position. At 298 K, R168 is always in the catalytic site, but the salt bridge is more labile as it is indicated by the distance increasing up to 8 Å, especially in subunits E, F, and G. This position of R168 in the apo state, with an extended lateral chain, mimics the one observed within the ternary complex. At 313 K, fluctuations increases. Remarkably, we observe that the lateral chain of R168 in subunit E exit the catalytic site during the last part of the simulation. This drastic local

change was not detected in others subunits. In subunit E, other structural reorganizations involving the kinked helix (H1G-H2G) and helix H2F, which carries R168, are observed. The mobile loop covering the catalytic site is not shown in the bottom panels of Figure S10. During the simulations, this loop always samples the open conformation for the higher temperatures T=298 K and T=313 K. We point out that in the snapshot at 313 K, H192 and D168 of the apo state display the same conformation as in the crystal structure.

Eukaryotic LDH are considered non-allosteric i.e. both the apo and the ternary complex look like the R-(active) state of bacterial LDH. Our data indicate that in certain conditions, fluctuations of the apo state of an eukaryotic LDH may sample some local conformations similar to those observed in the T-(inactive) state of allosteric bacterial LDH.

References

1. Torre, G. D. L., Huertas, J. M. L. & Carrasco, B. Calculation of hydrodynamic properties of globular proteins from their atomic-level structure. *Biophys. J* **78**, 719–730 (2000).

2. Clarke, A. R. *et al.* An investigation of the contribution made by the carboxylate group of an active site histidine-aspartate couple to binding and catalysis in lactate dehydrogenase. *Biochemistry* **27**, 1617–1622 (1988).

1 Figures SI

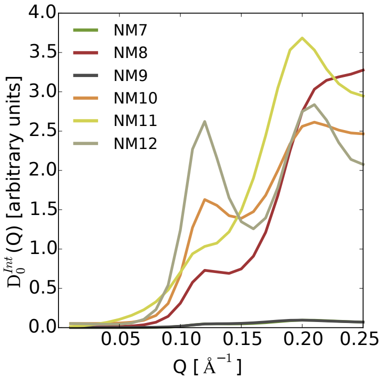

Figure S1. Contribution to internal diffusion from the first 6 nontrivial Normal Modes of the protein M5 LDH.

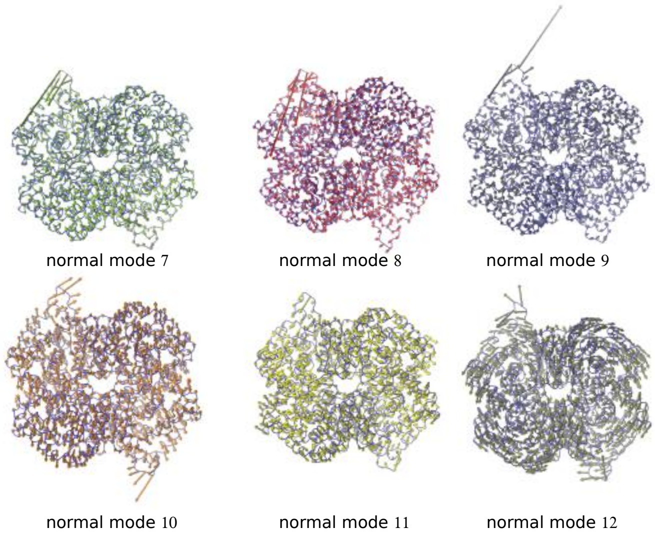

Figure S2. Representation of the first 6 nontrivial Normal Modes of the X-ray structure PBD 3H3F.

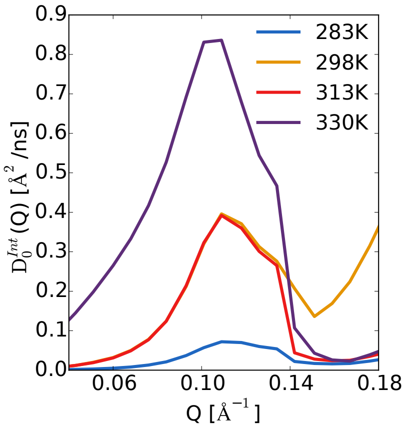

Figure S3. Contribution of the internal dynamics D_0^{int} to the diffusion spectra D_0.

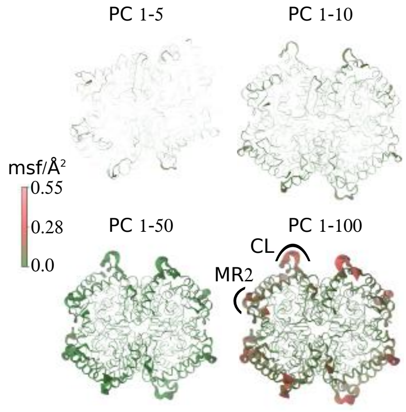

Figure S4. Mean squared fluctuations of C_α atoms of the protein calculated along the trajectories projected on a different set of principal component (PC) modes. The most flexible regions, namely the catalytic loop (CL) and the mobile region (MR2) are indicated by semicircular lines.

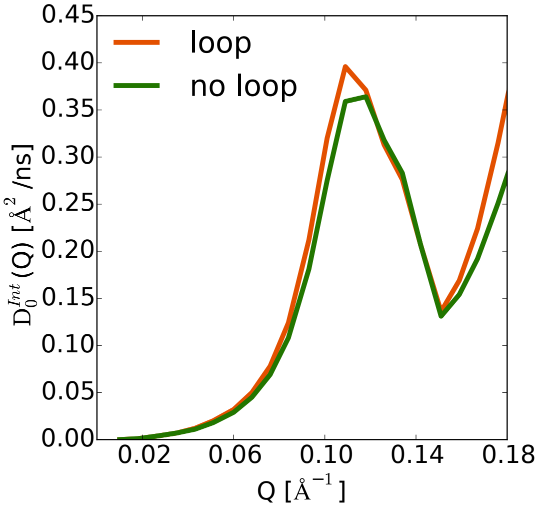

Figure S5. The internal dynamics diffusion coefficient extracted from fitting the $I(Q,t)/I(Q,0)$ from a single trajectory at T=298 K where the loop was removed ('no loop') and compared to that obtained from the standard trajectory ('loop').

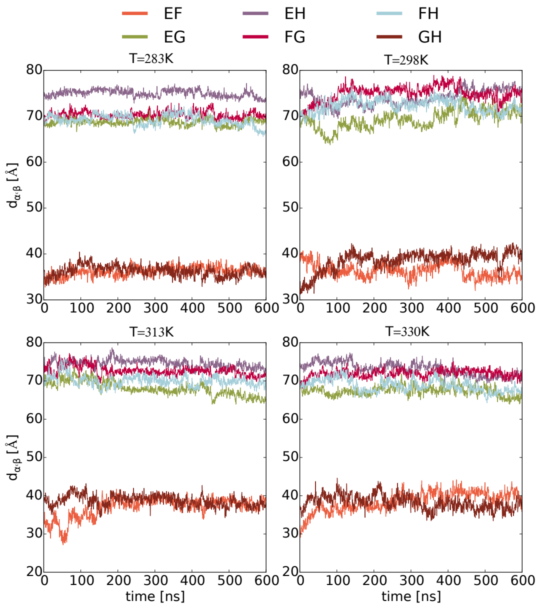

Figure S6. Timelines of inter-loop distances between four subunits named E, F, G, H, defined as $d_{\alpha,\beta} = (\frac{1}{N_\alpha}\frac{1}{N_\beta}\sum_i^{N_\alpha}\sum_j^{N_\beta}|r_i - r_j|^2)^{1/2}$.

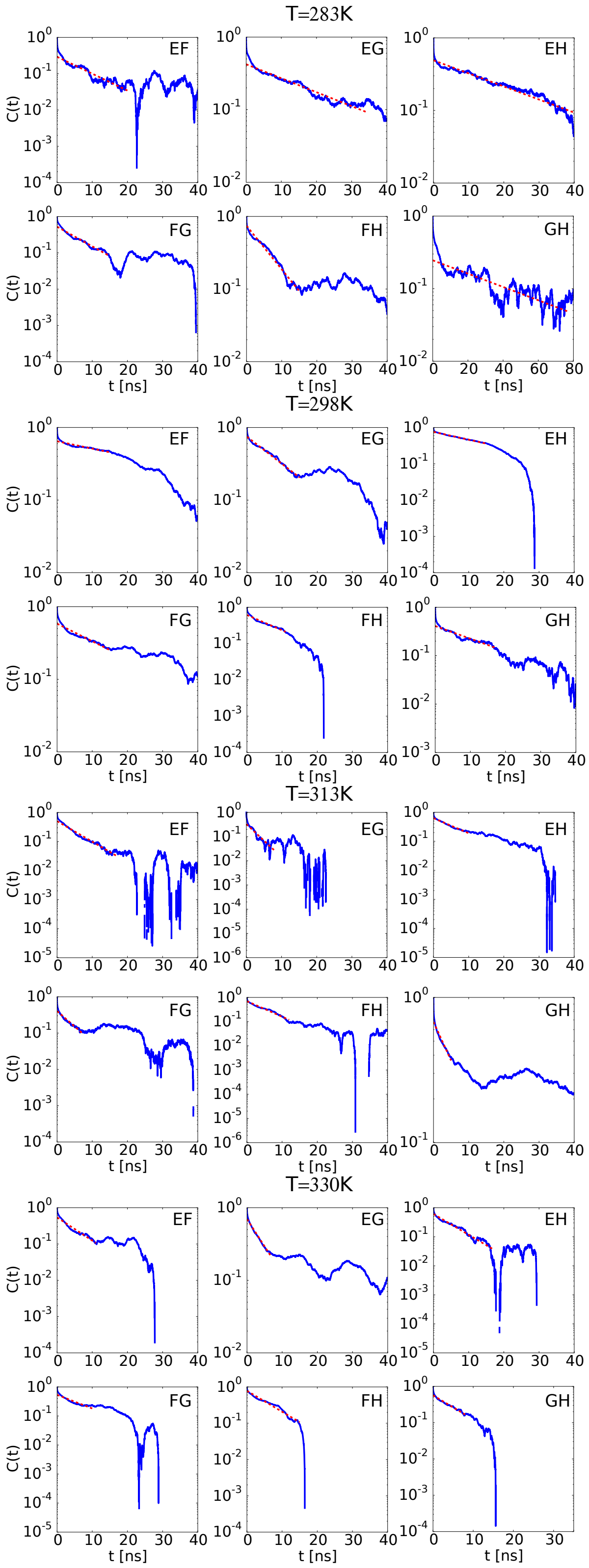

Figure S7. Time autocorrelation function, $C(t) =< d_{\alpha,\beta}(t) d_{\alpha,\beta}(0) > / < d_{\alpha,\beta}(0) d_{\alpha,\beta}(0) >$, of the interloop distances as defined by the metric $d_{\alpha,\beta} = (\frac{1}{N_\alpha N_\beta} \sum_i^{N_\alpha} \sum_j^{N_\beta} |r_i - r_j|^2)^{1/2}$. The relaxation time τ is obtained by fitting the first decay of the $C(t)$ by an exponential function. The characteristic time τ is used in the calculation of the diffusion coefficient in the harmonic approximation (see main text). The results are shown for all pairs of loops α, β in the four subunit protein, and for all simulated temperatures.

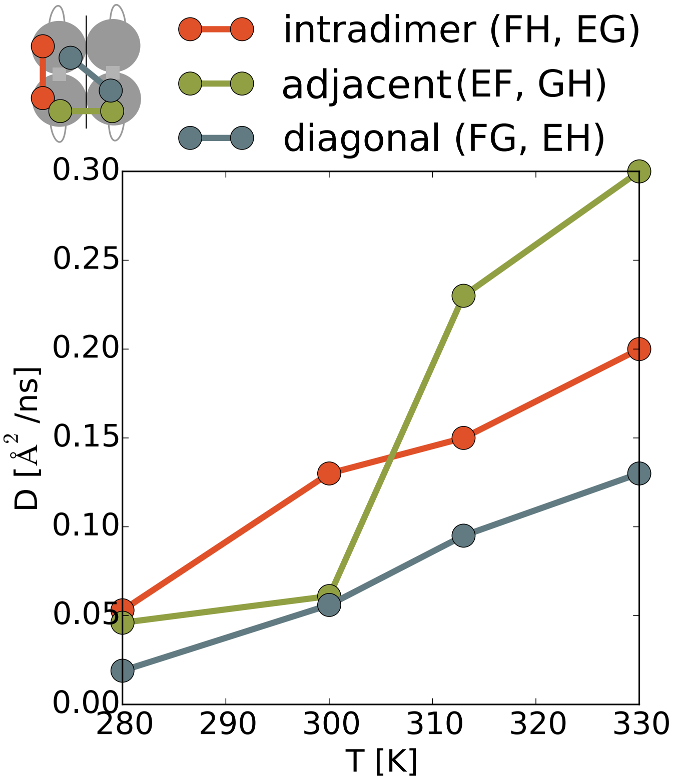

Figure S8. Diffusion coefficients calculated in the harmonic approximation for different inter-subunit loop distances and plotted as a function of temperature.

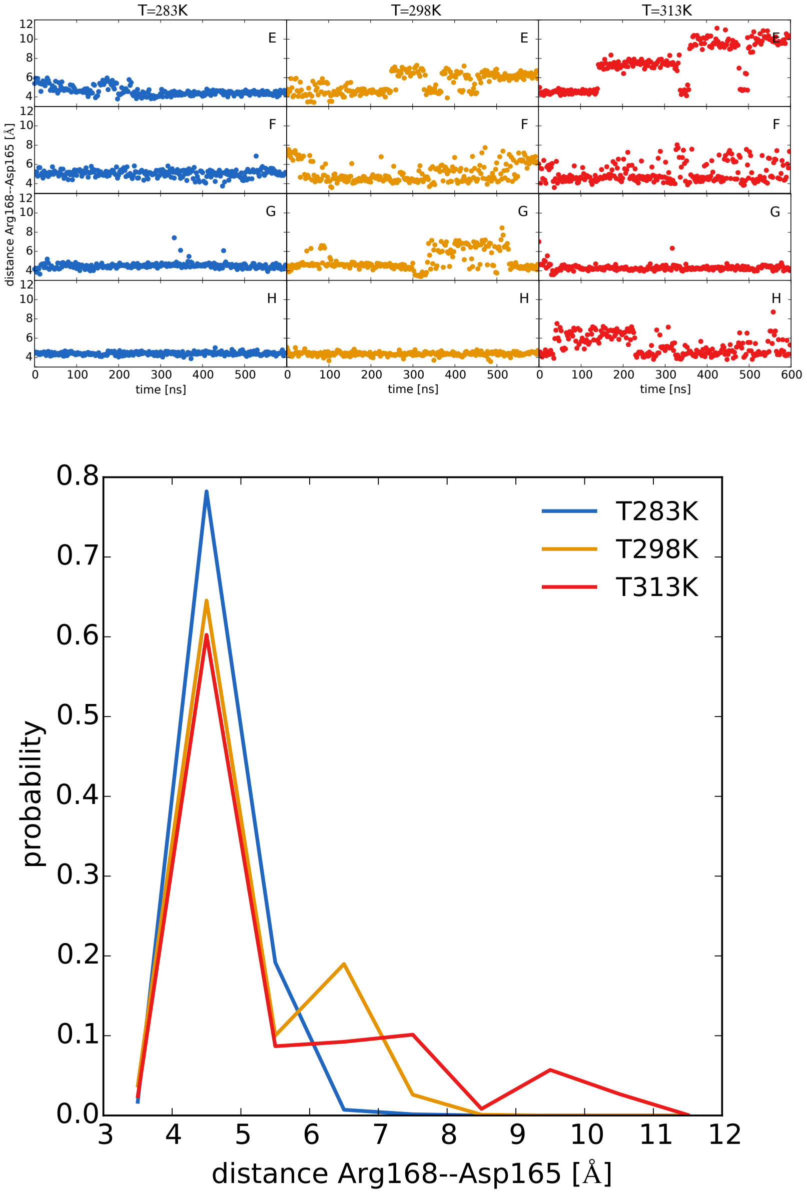

Figure S9. In the top panel, we report the evolution of the Arg168-Asp165 distance calculated for the four subunits (E, F, G, H) in the MD simulations at temperatures 283, 298, and 313 K. The probability distribution of the distances averaged over the four subunits is presented in the bottom panel, where the computed distances represent the center of mass of the charged terminals of the side chain ends.

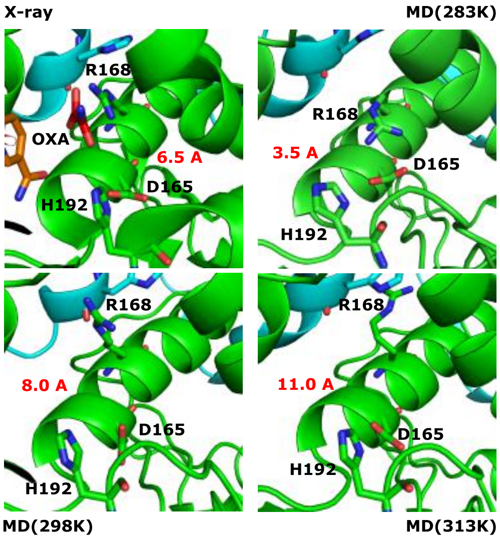

Figure S10. Representation of the different conformations sampled by the residue Arg168 in the active site during the MD simulations at different temperatures. In the top right panel we also report the organization of the catalytic site in the presence of oxamate and pyruvate molecules as resolved in the X-ray structure of the LDH of rabbit muscle 5 (PDB code 3F3H). For sake of comparison with Figure S9, we have also reported the instantaneous distance between the Arg168 and Asp165 charged terminals.

2 Tables SI

temperature	DLS [Å^2/ns]	HYDROPRO [Å^2/ns]
283 K	3.0	3.6
298 K	4.6	5.1
313 K	6.6	8.2

Table S1. Comparison between translational diffusion coefficients as measured by the Dynamic Light Scattering and calculated by the HYDROPRO program[1].

components summed	T=283 K [Å^2/ns]	T=298 K [Å^2/ns]	T=313 K [Å^2/ns]
1-5	0.0035 (4.99%)	0.028 (7.53%)	0.025 (6.99%)
1-10	0.014 (20.2%)	0.086 (23.3%)	0.077 (21.4%)
1-50	0.041 (59.3%)	0.22 (60.2%)	0.22 (61.3%)
1-100	0.050 (74.4%)	0.28 (74.2%)	0.28 (76.4%)

Table S2. Principal Component modes contribution to the internal dynamics at the first peak region ($Q = 0.118$ Å^{-1}).

temperature	N(t_{sim})	N_∞	τ (ns)	$N_{kinetic}$
283 K	8	23.0	1427.7	2
298 K	27	34.4	470.0	9
313 K	33	68.0	939.0	8
330 K	150	-	-	37

Table S3. Conformational and kinetic clustering of the MD simulations.

subunits involved	T=283 K [Å^2/ns]	T=298 K [Å^2/ns]	T=313 K [Å^2/ns]	T=330 K [Å^2/ns]
intradimer (FH, EG)	0.053	0.13	0.15	0.20
adjacent (EF, GH)	0.046	0.061	0.23	0.30
diagonal (FG EH)	0.019	0.056	0.095	0.13

Table S4. Diffusion coefficient of the interloop distances obtained as $D_{\alpha,\beta} = \frac{\langle \delta d^2_{\alpha,\beta} \rangle}{\tau}$, where d is the interloop distance defined for all atoms in the loop as $d_{\alpha,\beta} = (\frac{1}{N_\alpha}\frac{1}{N_\beta}\sum_i^{N_\alpha}\sum_j^{N_\beta}|r_i - r_j|^2)^{1/2}$, and τ is the relaxation time of the autocorrelation function of this distance.
